# Fouling Mitigation and Wastewater Treatment Enhancement through the Application of an Electro Moving Bed Membrane Bioreactor (eMB-MBR)

**DOI:** 10.3390/membranes8040116

**Published:** 2018-11-22

**Authors:** Jessa Marie J. Millanar-Marfa, Laura Borea, Mark Daniel G. de Luna, Florencio C. Ballesteros, Vincenzo Belgiorno, Vincenzo Naddeo

**Affiliations:** 1 Environmental Engineering Program, National Graduate School of Engineering, University of the Philippines, 1101 Diliman, Quezon City, Philippines; jmmillanar@gmail.com (J.M.J.M.-M.); mgdeluna@up.edu.ph (M.D.G.d.L.); fcballesteros@up.edu.ph (F.C.B.J.); 2 Sanitary Environmental Engineering Division (SEED), Department of Civil Engineering, University of Salerno, 84084 Fisciano, Italy; v.belgiorno@unisa.it (V.B.); vnaddeo@unisa.it (V.N.); 3 Department of Chemical Engineering, University of the Philippines, 1101 Diliman, Quezon City, Philippines

**Keywords:** electrochemical processes, fouling precursors, moving bed biofilm reactor, MBBR, voltage gradient

## Abstract

High operational cost due to membrane fouling propensity remains a major drawback for the widespread application of membrane bioreactor (MBR) technology. As a result, studies on membrane fouling mitigation through the application of integrated processes have been widely explored. In this work, the combined application of electrochemical processes and moving bed biofilm reactor (MBBR) technology within an MBR at laboratory scale was performed by applying an intermittent voltage of 3 V/cm to a reactor filled with 30% carriers. The treatment efficiency of the electro moving bed membrane bioreactor (eMB-MBR) technology in terms of ammonium nitrogen (NH_4_-N) and orthophosphate (PO_4_-P) removal significantly improved from 49.8% and 76.7% in the moving bed membrane bioreactor (MB-MBR) control system to 55% and 98.7% in the eMB-MBR, respectively. Additionally, concentrations of known fouling precursors and membrane fouling rate were noticeably lower in the eMB-MBR system as compared to the control system. Hence, this study successfully demonstrated an innovative and effective technology (i.e., eMB-MBR) to improve MBR performance in terms of both conventional contaminant removal and fouling mitigation.

## 1. Introduction

Membrane bioreactor (MBR) technology which involves the integration of membrane filtration with biological treatment has been widely investigated and deemed as a promising alternative to conventional wastewater treatment due to its numerous advantages [1,2,3]. However, the hydrophobic nature of commonly manufactured membranes makes it prone to membrane fouling which negatively impacts MBR efficiency [4] and increases operational and maintenance (O&M) costs. Approximately 34% of O&M costs are attributed to the energy requirement, mainly for pumping and the aeration of MBR for fouling mitigation, and roughly 28% of the costs are attributed to membrane replacement [2,5]. Because of the above, an effective process for fouling mitigation in MBR technology is necessary to expand its applications [6,7]. 

Numerous methods have been explored by research studies to address membrane fouling in MBRs. Some of these include membrane surface modification [8], the addition of adsorbents and coagulants [9,10,11], the addition of bio-carriers [12], and the application of an electric field and the application of ultrasound [7,13,14,15]. Among these, the application of an electric field to MBR reactors [4,16,17,18] and the addition of carriers to mimic a moving bed biofilm reactor (MBBR) [19,20,21,22] have gained increasing interest for membrane fouling control and wastewater treatment performance enhancement since these methods do not involve the addition of chemicals that may alter the activity inside the bioreactor and can be easily done and controlled in situ.

Electrochemical processes such as electrocoagulation, electro-osmosis and electrophoresis arising from electric field application in MBRs have been found effective in fouling mitigation and the enhancement of nutrient and contaminant removal from wastewater [4,23,24]. A study by Ibeid et al. [25] found that the application of an electric field on a pilot scale MBR could reduce the fouling rate up to x3-fold, whereas Hua et al. [4] observed a 7.8-fold reduction in the membrane fouling rate. Another study by Liu et al. [13] observed an increase in the filtration cycle from 11 days to 24 days when 1 mA current was applied. In terms of effluent quality, Bani-Melhem and Elektorowicz [26] noted an improvement in the chemical oxygen demand (COD) and PO_4_-P removal of 96% and 98%, respectively, upon intermittent electric field application, whereas Tafti et al. [27] noted 4% and 43% increases in COD and phosphate removals, respectively. The reduction of membrane fouling precursors, the improvement of sludge settleability and filterability, the limitation of growth of filamentous bacteria, and the promotion of conditions that favor nutrient and organic contaminant removal are some of the observed effects of electric field application that contribute to its better performance compared to a conventional MBR [4,9,23,28].

On the other hand, the moving bed membrane bioreactor (MB-MBR) has improved the treatment efficiency of the MBR by providing a higher surface area where growth and diversity of microorganisms are favored [19,21]. Furthermore, the addition of media in the form of carriers has promoted nutrient removal by allowing both aerobic, anoxic, and anaerobic conditions to spontaneously occur inside the carrier [29]. Mannina et al. [22] obtained high COD, nitrogen, and phosphorus removals of 98%, 53–69%, and 67–87%, respectively, for an MB-MBR system with C/N ratio of 10 and 5, whereas Subtil et al. [30] obtained 98% and 73% removal of ammonia and total nitrogen, respectively. Furthermore, a decrease of 6% in the membrane fouling rate was also noted in their study through the reduction of suspended solids in the reactor and the reduction of solids accumulation on the membrane surface by the attachment of biomass to the added carriers [12]. A review by Kawan et al. [21] noted that approximately 90% of solids in the reactor could be attached to the carrier which led to a decrease in membrane fouling and promoted a better environment for microbial growth [20,21,31].

With the known advantages of electric field application and the MBBR process, this work aimed to propose an innovative technology for fouling mitigation and treatment performance improvement of MBR through the integration of electric field application within an MBBR into an MBR at laboratory scale. The performance of this new technology, called electro moving bed membrane bioreactor or eMB-MBR, will be evaluated and compared to the MB-MBR control system.

## 2. Materials and Methods

### 2.1. Reactor and Materials

**Figure 1:**
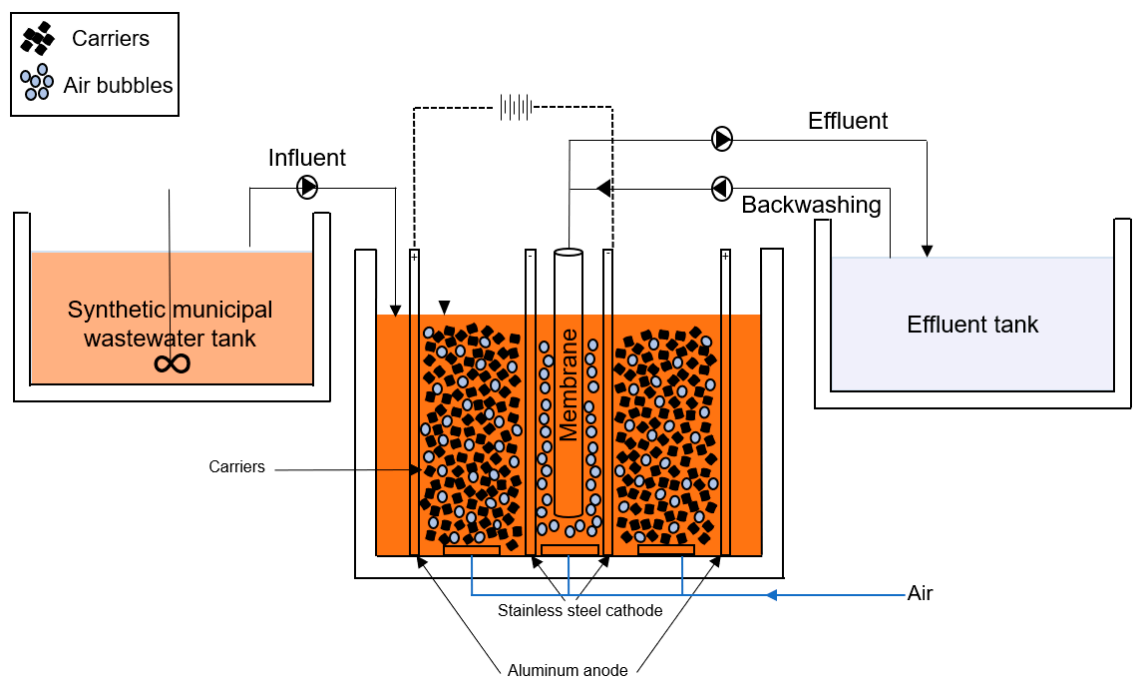
Experimental setup of the electro moving bed membrane bioreactor (eMB-MBR) system.

The experimental setup used in this study is shown in Figure 1. The reactor worked in two successive runs, each lasting for approximately 30 days: in the first run as an MB-MBR reactor and in the second run as an eMB-MBR reactor with the application of electrochemical processes. The membrane module used for all the runs was the ZeeWeed^®^-1 (ZW-1, Zenon Europe Kft, Oroszlany, Hungary) submerged polyvinylidene flouride (PVDF) hollow fiber ultrafiltration membrane module with a nominal pore diameter of 0.04 µm and an effective membrane surface area of 0.047 m^2^ placed in the center of the reactor. The MB-MBR setup was obtained by using a cylindrical reactor with a working volume of 13 L filled with BIOMASTER BCN 012 KLS Amitec^®^ carriers (Amitec, Cernusco sul Naviglio, Italy), with a filling ratio (FR) of 30% and a net surface area of 500 m^2^/m^3^. This setup was referred to as the control system. On the other hand, the eMB-MBR setup used the same reactor and membrane. A cylindrical aluminum anode and a stainless steel cathode connected to a digital external DC power supply (CPX400, TTi, 0–60 V, 0–20 A) were placed around the membrane module with a radial distance of 6 cm from each other. Dissolved oxygen (DO) concentration inside the bioreactor was maintained using aerators placed at the bottom. Good mixing and slight air scouring were also promoted by the installed aeration system, as is shown in Figure 1. Synthetic municipal wastewater with characteristics described in Borea et al. [32] was continuously fed to both bioreactors and treated water was pumped at a constant flux of 15 LMH. Mixed liquor volatile suspended solids (MLVSS) during the experiment were 2700 to 4000 mg/L, whereas the hydraulic retention time (HRT) used was 18 h and the solids retention time (SRT) was approximately 40 days.

The control system (MB-MBR) was operated with the electrodes disconnected from the power supply, whereas the eMB-MBR system was operated with an intermittent application of 3 V/cm at an operation mode of 5 min ON and 20 min OFF using an electronic controller referred to in previous studies [23,24,25,32]. 

### 2.2. Analytical Methods

The pH, dissolved oxygen (DO) concentration, temperature, and redox potential inside the bioreactor were monitored using a multiparametric probe (Hanna Instruments, Padova, Italy, HI2838). Samples were obtained from the influent, effluent, and supernatant. Concentrations of COD and nutrients (ammonium nitrogen (NH_4_-N), nitrate nitrogen (NO_3_-N), nitrite nitrogen (NO_2_-N) and orthophosphate (PO_4_-P)) from each sample were measured according to the standard methods [33]. Anions in effluent and the bioreactor were determined by filtering samples from the permeate and mixed liquor, respectively, and using an ion chromatograph. The percent removals of COD, NH_4_-N, and PO_4_-P were analyzed as removals from biodegradation, filtration, and total removal. Total percent removal was computed using Equation (1), whereas biodegradation removal for the MB-MBR and biodegradation with electrocoagulation removal for the eMB-MBR were computed from the difference in influent and reactor concentrations using Equation (2). Filtration removal was the difference between the total removal and biodegradation removal computed by using Equation (3).

(1) $$\mathrm{Total}\;\mathrm{removal}\;\left[\%\right]=\frac{C_{i}-C_{e}}{C_{i}}\times 100\%$$

(2) $$\mathrm{Biodegradation}\;\mathrm{or}\;\mathrm{Biodegradation}+\mathrm{Electrocoagulation}\;\mathrm{removal}\;\left[\%\right]=\frac{C_{i}-C_{r}}{C_{i}}\text{×}100\%$$

(3) Filtration removal [%]=Total removal [%]−Biodegradation removal [%]

Mixed liquor suspended solids (MLSS) and mixed liquor volatile suspended solids (MLVSS) inside the bioreactor were measured using standard methods [33]. Biofilm solids (BS) were measured using Plattes et al. [34] as reference. Membrane fouling inside the bioreactor was monitored by measuring the transmembrane pressure variation over time, through a pressure transducer (PX409-0-15VI, Omega, Sunbury, OH, USA) connected to a datalogger (34972A LXI Data Acquisition/Switch unit, Agilent, Malaysia), the concentrations of known membrane fouling precursors, namely extracellular polymeric substances (EPS), soluble microbial products (SMP), and transparent exopolymeric particles (TEP). EPS and SMP were classified into proteins (EPSp, SMPp) and carbohydrates (EPSc, SMPc) [35,36,37]. A heating method was used to separately obtain EPS and SMP samples from sludge flocs. Mixed liquor samples were filtered, and the filtrate obtained was referred to as the SMP. After SMP extraction, the remaining samples were filled with deionized water and heated inside an oven at 80 °C. These samples were once again filtered and the filtrate from this process was referred to as the EPS [6,38]. Photometric methods from Frølund et al. and DuBois et al. were then used to analyze both the protein and carbohydrates components of EPS and SMP [6,35,36,39], respectively, using bovine serum albumin (BSA) (Sigma, St. Louis, MO, USA) and D-glucose (Sigma, St. Louis, MO, USA) as standards. A method used and developed in a previous study [23] was used to analyze the TEP concentration. The concentration of TEP, EPS, and SMP, in terms of protein (EPSp, SMPp) and carbohydrate (EPSc, SMPc) was then normalized by the MLVSS content. Fouling precursor concentrations and transmembrane pressure (TMP) values were then correlated.

## 3. Results and Discussion

### 3.1. COD, Nutrients, and Fouling Precursor Behavior inside the Bioreactor

#### 3.1.1. MB-MBR System

**Figure 2:**
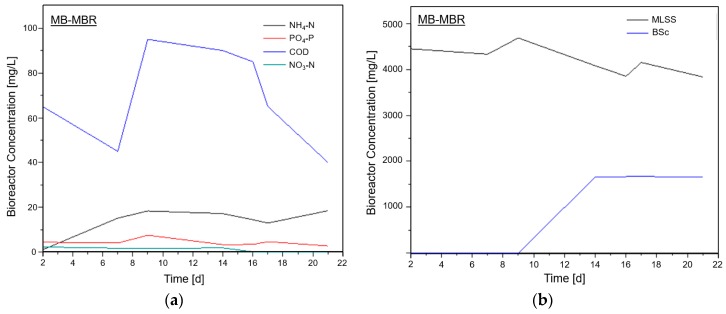
(**a**) Chemical oxygen demand (COD), nutrient, and (**b**) solid concentration inside the moving bed membrane bioreactor (MB-MBR).

Figure 2a shows the behavior of COD, NH_4_-N, NO_3_-N, and PO_4_-P concentrations, whereas Figure 2b shows the trends of MLSS and biofilm solid concentration on the carriers (BSc) over time inside the MB-MBR system.

These plots can be divided into five parts. During the first part (days 3 to 7), it was observed that the setup was still in the stabilization stage where microorganisms started to acclimatize [40]. However, a high decrease in COD could be due to the high concentration of readily biodegradable COD in the feed wastewater [23]. NH_4_-N nitrification during this part was limited by slow growing nitrifying bacteria [41]; thus, the addition of influent NH_4_-N resulted in an increase in NH_4_-N concentration inside the bioreactor. At this stage, MLSS and BSc also did not undergo significant changes in concentration which indicated that there was no remarkable increase in microbial community or activity inside the bioreactor, justifying the occurrence of stabilization inside the bioreactor [20]. During the second part (days 7 to 9), no significant increase was observed with MLSS and BSc concentration inside the bioreactor. Additionally, increasing concentrations of COD and NH_4_-N were observed due to the additional influent COD and NH_4_-N, indicating a minimal biodegradation that could occur during the stabilization stage. An upward trend in PO_4_-P concentration signaled phosphate accumulating organism (PAO) activity inside the reactor that could suggest a slight change in operating conditions inside the bioreactor or inside the carriers [29]. At days 9 to 14 (third part), the setup started to become more active. Although there was a decrease in MLSS concentration at this stage, a sharp increase in BSc was observed. The decrease in MLSS in the reactor could be attributed to the number of solids that were attached to the carriers [20]. In addition, higher microbial community and activity was indicated by the increase in BSc [30]. This resulted in the decrease in COD, NH_4_-N and PO_4_-P concentrations. Because of the supposedly high microbial activity and growth in the previous stage, and as the system approached days 14 to 17 (fourth part), a sharp decrease in COD was observed, implying that the majority of available DO was consumed by COD. This resulted in a low nitrification rate and a higher PO_4_-P concentration. MLSS and BSc plots appeared flat at this stage, signaling that there was no significant microbial activity brought about by population shift (e.g., aerobic to anaerobic organisms). Finally, at days 17 onwards (fifth part), the decreasing trend of COD concentration was not as sharp as days 14 to 17, implying a lower biodegradation rate. The nitrification process was also hampered. These could be attributed to anoxic conditions exhibited by the inner part of the carrier with deposited biofilm [42]. After this stage, the bioreactor started to stabilize, and no significant changes were noted.

**Figure 3:**
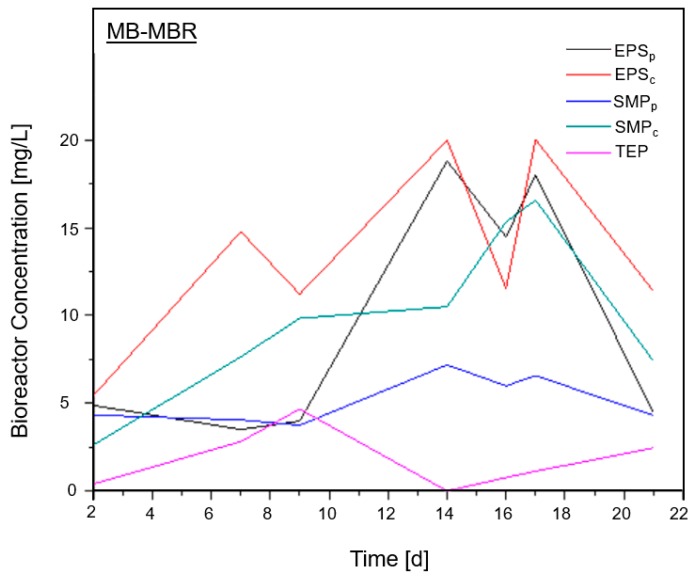
Fouling precursor concentration inside the MB-MBR.

Since fouling precursors are products of microbial activity and decay, fluctuation in the concentrations of these compounds might signal that an operating condition-driven change in microbial activity occurred [9]. Figure 3 shows the plots of EPS, SMP, and TEP concentration over time with its changing trend complementing the earlier findings.

#### 3.1.2. eMB-MBR System

**Figure 4:**
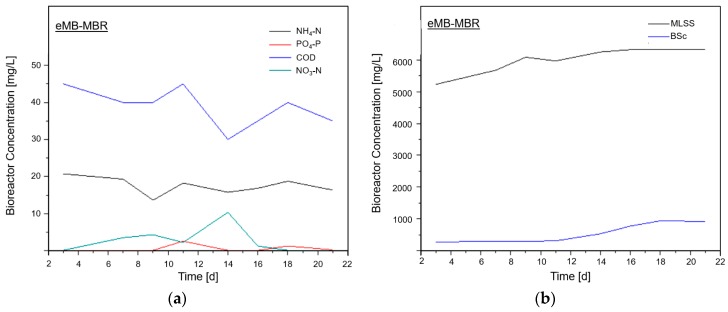
(**a**) COD, nutrient, and (**b**) solid concentration inside the eMB-MBR.

Figure 4 shows the plots for COD, nutrients, and solids behavior inside the eMB-MBR system. From Figure 4a, COD, PO_4_-P, and NH_4_-N all have almost no change in concentrations. This could be due to the impact of the electric field application on the system. The COD content of the synthetic wastewater used was readily biodegradable; thus, it already had high removal even at the start of the experiment [23]. Additionally, PO_4_-P also had a high removal rate due to the combined effects of biodegradation and electrocoagulation [24], whereas NH_4_-N removal was still dominated by biodegradation via the alternating aerobic and anaerobic process with minimal effects of electrocoagulation [23]. MLSS and BSc concentration plots followed an upward trend since energy is generated during substrate catabolism under anaerobic or aerobic conditions [22].

The eMB-MBR system was divided into four parts. The first part (days 3 to 9) corresponded to a slow increase in MLSS and BSc, indicating the occurrence of a stabilization stage inside the bioreactor [40]. It is proposed that the reactions in the bioreactor were dominated by the effect caused by the electric field application, indicated by the removal of COD and NH_4_-N with the increase in NO_3_-N even during the stabilization stage. No increase in PO_4_-P concentration was observed owing to reduced activity of PAO under aerobic conditions. The same result was observed by García-Gómez et al. [14]. PO_4_-P removal was attributed to electrocoagulation [43]. During days 9 to 11 (second part), a lower MLSS concentration and an almost constant BSc concentration were noted. Lower energy was harnessed by microorganisms under the governing conditions, resulting in a lower cellular mass being synthesized and leading to the increase in COD, PO_4_-P, and NH_4_-N concentrations and the decrease in NO_3_-N concentration [44]. It is proposed that, at that time, the bioreactor approached an anoxic stage induced by the electric field application. This condition could be seen from the activity of PAO and the inhibition of the nitrification process. Moreover, days 11 to 14 (third part) had a noticeable increase in MLSS and BSc concentrations, as is shown in Figure 4b. The combination of the aerobic biodegradation process with the electric field application led to a high COD removal rate and a lower NH_4_-N and PO_4_-P concentration. Finally, at day 16 onwards (fourth part), an increase in MLSS and BSc concentrations was still observed. However, the high removal rate of COD observed in days 11 to 14 might have consumed the available DO and then the NO_3_-N concentration flattened, showing very little occurrence of nitrification.

**Figure 5:**
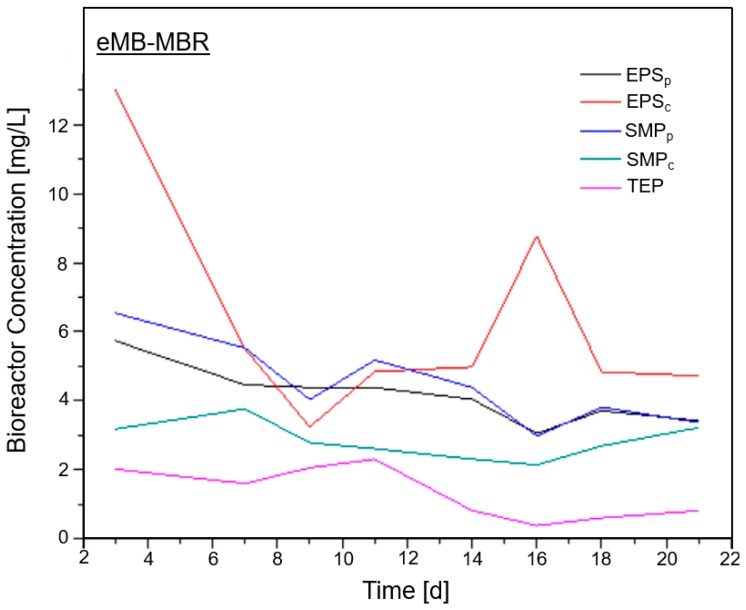
Fouling precursor concentration inside the eMB-MBR.

The proposed shift in the operating conditions caused by the electric field application might have resulted in the change in EPSp, SMP and TEP concentrations shown in Figure 5.

Based on the observations made about the MB-MBR and eMB-MBR systems’ performance, it was established that microorganisms significantly affect MB-MBR and eMB-MBR systems. These microbial communities are affected by operating conditions inside the bioreactor. Hence, future studies should note the19-docosahexaenoic acid (DHA) and the specific oxygen uptake rate (SOUR) to have a better understanding of the microbial community behavior inside the bioreactor.

### 3.2. Removal of Nutrients and Chemical Oxygen Demand

**Figure 6:**
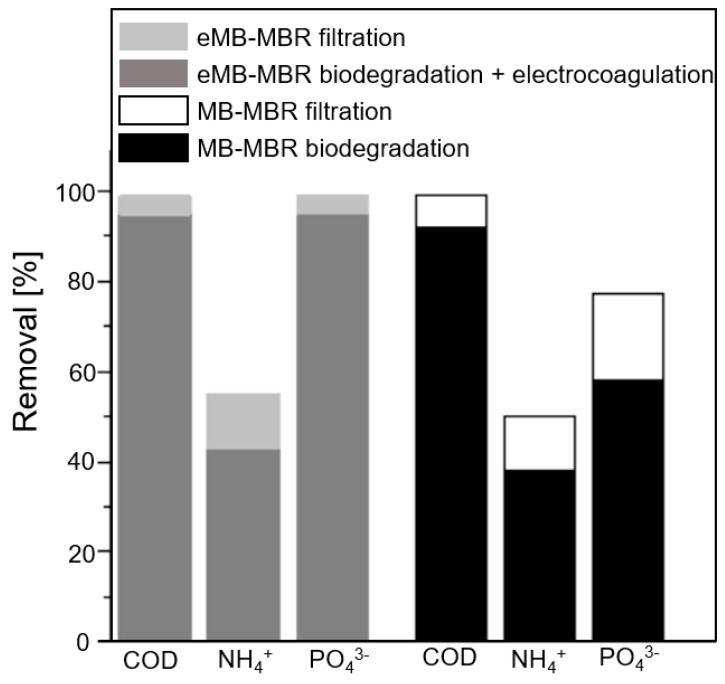
COD, NH_4_^+^ and PO_4_^3−^ removal efficiencies by MB-MBR and eMB-MBR configurations.

The combination of the electric field application with freely moving carriers inside the bioreactor enhanced the nutrient removal. As can be seen in Figure 6, an improvement of 5.2% and 22% was observed for NH_4_-N and PO_4_-P removal efficiencies, respectively, in the eMB-MBR system compared to the MB-MBR system. 

NH_4_^+^ removal in MB-MBR systems can be limited by the concentrations and diffusion rates of both dissolved oxygen (DO) and NH_4_^+^ in the moving bed [29]. Thus, the low NH_4_^+^ removal in the MB-MBR system can be attributed to insufficient aerobic sites inside the carriers. Nevertheless, the applied current in the eMB-MBR system controlled several electrochemical mechanisms inside the bioreactor. At the anode side of the reactor, NH_4_-N concentration decreased due to the oxidation to NO_3_-N [45,46], whereas at the cathode side, reductive reactions predominated. These reactions consumed DO and subsequently induced anoxic conditions inside the bioreactor with the application of the electric field [32]. Alternating anoxic and aerobic conditions inside the carriers and induced by electric field application favored the occurrence of the nitrification process, causing higher NH_4_^+^ removals in the eMB-MBR which corresponds with the results from previous studies [46,47]. Additionally, since DO concentration has a significant impact on microbial activity, the alternation of aerobic and anoxic conditions enhanced the NH_4_-N removal in the eMB-MBR in terms of biodegradation following the nitrification and denitrification equations shown in Equations (4) and (5). The occurrence of the nitrification process was observed from the increase in NO_3_-N average concentrations from 0.10 mg/L in the influent to 1.61 mg/L in the reactor, whereas the occurrence of the denitrification process was noted from the higher NO_3_-N average concentration (1.61 mg/L) inside the reactor compared to the effluent average NO_3_-N concentration (1.19 mg/L), signifying that nitrate was reduced to nitrogen gas. On the other hand, the higher attraction of sludge particles to the membrane module due to the absence of an electric field in the MB-MBR system facilitated the formation of a secondary layer that was both biologically active and could serve as a good filtration medium. Thus, higher NH_4_-N removal in terms of filtration in the MB-MBR system is attributed to the additional filtration and biodegradation that might have occurred when water passed through this secondary layer.

NH_4_^+^ + 2O_2_ → NO_3_^−^ + 2H^+^ + H_2_O (4)

NO_3_^−^ →NO_2_^−^ → NO → N_2_O → N_2(g)_ (5)

In addition, the electric current flowing in the eMB-MBR system released Al^3+^ and Al_6_(OH)_15_^3+^ ions from the dissolution of aluminum anode and formed H_2_ and OH^−^ from water at the cathode side. These chemical species combined with orthophosphate ions to form AlPO_4(s)_ and [Al_6_(OH)_15_]PO_4(s)_ compounds according to Equations (6) and (7) [16,38]. Moreover, as compared to the Fe anode, the aluminum hydroxide species formed from the aluminum anode were reported to have a higher surface area and were more efficient in terms of particle entrapment and adsorption then the iron hydroxide formed from the Fe anode [16]. Thus, 98.7 ± 0.2% average total PO_4_-P removal was achieved in the eMB-MBR compared to 76.7 ± 14.1% average total PO_4_-P removal in the MB-MBR system. The occurrence of the electrocoagulation process was corroborated from the decrease in weight of the aluminum anode from 349 g at the start of the experiment to 306 g at the end of the experiment. Nevertheless, the PO_4_-P filtration performance of the MB-MBR was higher than that of the eMB-MBR, having filtration removals of 19.2 ± 13.4% and 3.9 ± 10.9%, respectively. The reason was that only a small concentration of PO_4_-P was available for filtration since most of it was removed by the combination of biological and electrocoagulation processes in the eMB-MBR system. Additionally, as discussed in NH_4_-N removal, the newly produced dynamic membrane might have contributed to a better filtration performance of the remaining PO_4_^3^-P in the MB-MBR system.

Al^3+^ + PO_4_^3−^ → AlPO_4(s)_ (6)

Al_6_(OH)_15_^3+^ + PO_4_^3−^ → [Al_6_(OH)_15_]PO_4(s)_ (7)

From Figure 6, high total COD removals were noted for both systems at 98.6% and 98.7% for the MB-MBR and eMB-MBR systems, respectively. Of the COD removal efficiencies presented, approximately 4–7% was attributed to filtration and more than 90% to biodegradation. The high COD removal efficiencies of the two MBR systems are directly linked to the highly biodegradable sucrose and glucose content of the synthetic municipal wastewater.

### 3.3. Control of Membrane Fouling

**Figure 7:**
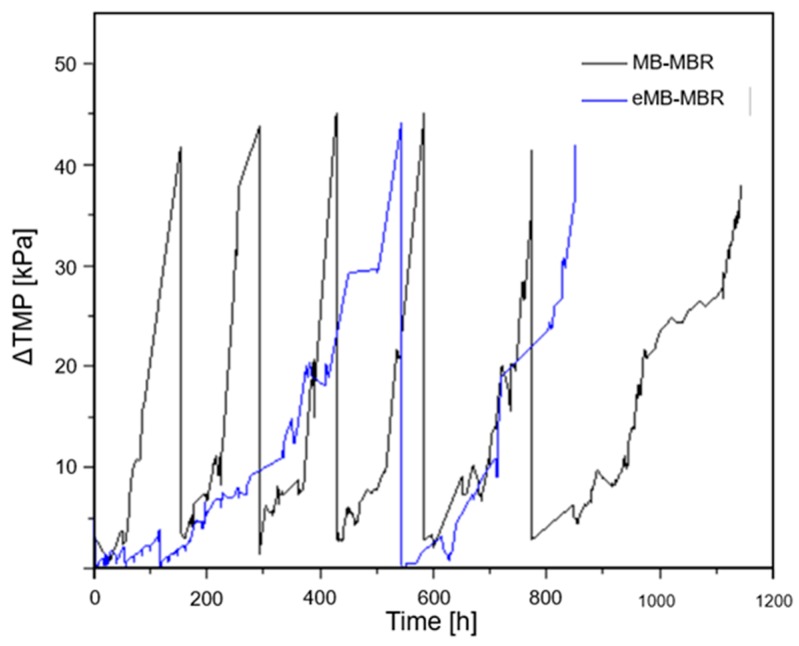
Comparison of Δ transmembrane pressure (TMP) of the MB-MBR and the eMB-MBR over time.

Membrane fouling is one of the major challenges in MBR technology implementation. Fouling is characterized by a rising TMP and the simultaneous flux decline resulting from the build-up of extracellular organics, microbial cells, solid particles, and other inorganic materials on the membrane surface [17,35]. One simple method to control fouling is by chemical cleaning of the membrane; thus, each sudden drop in TMP corresponds to the application of chemical cleaning on the fouled membrane. In this study, it was observed that less frequent chemical cleaning was required for the eMB-MBR system compared to the MB-MBR system. Additionally, a longer filtration cycle and a significant decrease in membrane fouling rate (60%) were obtained when an electric field was applied, as is shown in Figure 7. This result suggests that electric field application positively affects membrane fouling mitigation inside the bioreactor. This finding is consistent with those reported in previous studies whereby the application of an electric current to the bioreactor caused lower fouling rates and subsequently less frequent membrane cleaning [4,23,25,29,45,48]. Thus, effective fouling control in the eMB-MBR system enabled the membrane to operate at longer periods prior to chemical cleaning.

As MBR technology mainly relies on microbial activity for biodegradation, microorganisms are considered a vital key to MBR applications. Nonetheless, microbial activity such as substrate metabolism and microbial decomposition produce fouling precursors inside the membrane bioreactor. These include EPS and SMP that are principally made of proteins and high molecular weight carbohydrates and TEP. Based on some studies, proteins commonly form non-covalent networks that cause severe membrane fouling, whereas high molecular weight carbohydrates such as polysaccharides are difficult to mineralize and tend to behave like gel at acidic and neutral pH. Moreover, TEP are gel-like biopolymers that easily adsorb on the membrane surface and increase sludge viscosity [23,24,47]. Thus, to optimize the role of microorganisms, it is noteworthy to monitor the concentrations of these fouling precursors along with TMP and to minimize their respective concentrations inside the system.

**Figure 8:**
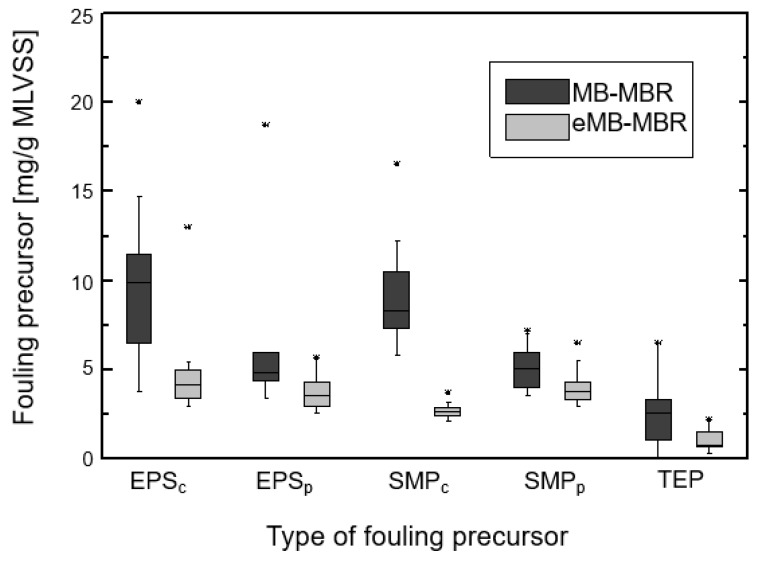
Fouling precursor concentrations in the MB-MBR and eMB-MBR systems.

The effect of electric field application was investigated through the concentrations of major fouling precursors such as EPS, SMP, and TEP. Figure 8 represents a box plot with maximum fouling precursor concentration and standard deviation over the period of operation. From the plots in Figure 8, it can be observed that lower concentrations of EPS, SMP, and TEP were obtained in the eMB-MBR system as compared to the MB-MBR.

Electric field application decreased the membrane fouling rate and decreased the transmembrane pressure (TMP) rise in the eMB-MBR via electrochemical processes. The electrocoagulation process produced positively charged metal ions resulting in foulant attraction, neutralization, and destabilization. Additionally, positively charged Al^3+^ and Al_6_(OH)_15_^3+^ ions reduced repulsive forces between flocs, leading to an increase in floc size and a lesser floc deposition on the membrane surface. Moreover, electrophoresis and electro-osmosis processes drove the negatively charged foulants away from the membrane and moved the positively charged bulk liquid toward the cathode and, thus, the membrane [17,38,46].

Moreover, an increase in the biosolid concentration on the carriers illustrated in Figure 2 and Figure 4 suggest that the carriers inside the bioreactor serve as attachment media to decrease the amount of biosolids that may accumulate on the membrane surface, resulting in a lower filtration resistance. The same was reported by Chen et al. [12]

Humic substances are strongly hydrophobic substances frequently associated with EPS that are contributors to membrane fouling. Based on some studies, these substances modify the membrane surface through their combination with proteins and polysaccharides via hydrophobic and electrostatic interactions and adsorption on the membrane surface [13,49]. Thus, low concentrations of these substances are preferred in the system. For this experiment, the average of the difference in influent and effluent humic substance concentration was expressed as the removal. A higher removal of humic substances computed from UV_254_ absorbance was obtained for the eMB-MBR system (92.36 ± 12.6% removal) than for the MB-MBR system (86.8 ± 40.2% removal).

The concentrations of EPS and SMP were investigated using protein and carbohydrate concentrations. The results shown in Figure 8 illustrate the positive impact of electric field application in the reduction of fouling precursor concentrations. EPS and SMP concentrations expressed as proteins and carbohydrates were noticeably lower in the eMB-MBR than in the MB-MBR. The application of an electric field induced the electrochemical oxidation of water and favored the formation of hydroxyl radicals. This resulted in the conversion of these fouling precursors into lower molecular weight compounds that are easier to mineralize [35,46,47]. Furthermore, electrocoagulation promoted the destabilization of foulants in suspension. Consequently, the reduction of SMP and TEP concentrations led to a less viscous sludge and cake layer, resulting in a higher filterability and a lower membrane resistance [25]. Lower fouling precursor concentrations in the eMB-MBR suggest that the applied electric field and the generated metal ions were not detrimental to microorganisms inside the system.

## 4. Conclusions

The results of this study showed that an effective and innovative technology called eMB-MBR was successfully obtained from the integration of electrochemical processes within an MBBR into an MBR reactor. This new technology was evaluated and compared to a control system (MB-MBR). An improvement in treatment efficiency especially in terms of nutrient removal and a significant reduction in fouling of approximately 60% were obtained for the eMB-MBR compared to the MB-MBR. Thus, eMB-MBR technology can be considered a promising new technology to further improve MBR and MBBR systems for wastewater treatment in terms of conventional contaminant removal and fouling mitigation.
